# Structural brain preservation: a potential bridge to future medical technologies

**DOI:** 10.3389/fmedt.2024.1400615

**Published:** 2024-09-09

**Authors:** Andrew T. McKenzie, Ariel Zeleznikow-Johnston, Jordan S. Sparks, Oge Nnadi, John Smart, Keith Wiley, Michael A. Cerullo, Aschwin de Wolf, Francesca Minerva, Ramón Risco, George M. Church, João Pedro de Magalhães, Emil F. Kendziorra

**Affiliations:** ^1^ Apex Neuroscience, Salem, OR, United States; ^2^ School of Psychological Sciences, Monash University, Melbourne, VIC, Australia; ^3^ Turner Institute for Brain and Mental Health, Monash University, Melbourne, VIC, Australia; ^4^ Brain Preservation Foundation, Ashburn, VA, United States; ^5^ Advanced Neural Biosciences, Portland, OR, United States; ^6^ Department of Philosophy, University of Milan, Milano, Italy; ^7^ Escuela Superior de Ingeniería, Universidad de Sevilla & National Accelerators Center, CNA-CSIC, Seville, Spain; ^8^ Department of Genetics, Harvard Medical School, Boston, MA, United States; ^9^ Wyss Institute for Biologically Inspired Engineering, Harvard University, Boston, MA, United States; ^10^ Institute of Inflammation and Ageing, University of Birmingham, Birmingham, United Kingdom; ^11^ Oxford Uehiro Centre for Practical Ethics, University of Oxford, Oxford, United Kingdom; ^12^ European Biostasis Foundation, Riehen, Switzerland

**Keywords:** brain preservation, biostasis, connectomics, brain perfusion, fluid preservation, molecular nanotechnology

## Abstract

When faced with the prospect of death, some people would prefer a form of long-term preservation that may allow them to be restored to healthy life in the future, if technology ever develops to the point that this is feasible and humane. Some believe that we may have the capacity to perform this type of experimental preservation today—although it has never been proven—using contemporary methods to preserve the structure of the brain. The idea is that the morphomolecular organization of the brain encodes the information required for psychological properties such as personality and long-term memories. If these structures in the brain can be maintained intact over time, this could theoretically provide a bridge to access restorative technologies in the future. To consider this hypothesis, we first describe possible metrics that can be used to assess structural brain preservation quality. We next explore several possible methods to preserve structural information in the brain, including the traditional cryonics method of cryopreservation, as well as aldehyde-stabilized cryopreservation and fluid preservation. We focus in-depth on fluid preservation, which relies on aldehyde fixation to induce chemical gel formation in a wide set of biomolecules and appears to be a cost-effective method. We describe two theoretical recovery technologies, alongside several of the ethical and legal complexities of brain preservation, all of which will require a prudent approach. We believe contemporary structural brain preservation methods have a non-negligible chance of allowing successful restoration in the future and that this deserves serious research efforts by the scientific community.

## Introduction

Many people desire to live longer in good health, feeling that they have yet to fully experience life, enjoy its pleasures, share moments with friends and family, and contribute to the world (1–6). However, given the inherent difficulties involved in halting or reversing biological aging, alongside relatively paltry societal investment in interventive gerontology (7), it is unlikely that aging will be sufficiently slowed in the next few decades to significantly extend maximum lifespan (8). On top of that, even if biological aging became preventable or reversible, there would always be injuries and diseases that are acutely lethal because treatment was not available or had not been invented yet.

One hypothetical option for an individual wishing to avoid an imminently fatal situation would be for the patient to undergo a *suspended animation* procedure. In suspended animation, a person's body would be preserved for the long-term in a way known to be able to be reversed at the time of one's choosing. However, despite some historical aspirations that it would be achievable soon (9–11), long-term suspended animation is not yet possible. The prospect of long-term suspended animation is still regarded with skepticism by the cryobiology research community because it is not yet possible to reversibly cryopreserve large organs such as the heart or brain, let alone an entire body (12, 13). It is worth noting that there is research ongoing in areas related to short-term states of suspended animation, such as torpor and hibernation, that may offer substantial mechanistic insights (14, 15). However, even if long-term suspended animation were developed, some people would not qualify for the initial procedures due to medical barriers. For example, the “no-reflow phenomenon” that occurs after cardiac arrest may prevent the complete perfusion of the brain (16, 17). This leaves a critical question for any acutely lethal condition now or in the future: What options exist when reversible suspended animation is not possible?

While demonstrably reversible suspended animation is not an option, a possible alternative is the *structural preservation* of the body, with the goal of retaining the molecular constituents of a person sufficiently intact for future repair and restoration. The simple idea here is that although reversible suspended animation does not currently exist, resuscitation procedures may be developed in the future, alongside technology to cure the acutely lethal condition, including trauma, ischemic injury, and chronic conditions such as biological aging (18–22).

Here, we will focus on the preservation of the brain. The predominant view among both philosophers and the general public is that a person survives over time through the continued existence of psychological properties that define their personal identity (23–26). As it is the brain which enables the continuity of memories, beliefs, personality, and other psychological properties across a person's lifespan, it is the crucial organ that must be preserved for a person to survive. We do not discount the potential value of additionally preserving the rest of the body, but in this paper, we limit our discussion to brain preservation, as it is the most essential organ for a person's survival.

## Experimental preservation with the potential for recovery

We can imagine a hypothetical procedure that would allow for brain preservation with *verifiable* preservation of a person's psychological properties. Such a procedure would be able to preserve the structural information that provides their memories, personality, cognition, and other valued aspects of their psychology. However, this proposal has three problems. First, we do not yet know what exactly the structural correlates of psychological properties are. While we can currently provide reasonable estimates, and our confidence in these is constantly improving as our knowledge of neuroscience improves, they remain estimates. For example, there is disagreement about which types of biomolecules are necessary to retain the information content required for long-term memory recall (27). Unknown unknowns will remain for this question, at least until we can reproducibly decode memories from static brains. Second, no one has yet published a demonstration of a procedure on humans that can reliably preserve the whole connectome with traceability intact. Finally, many would consider any strict threshold for verifiable brain preservation too conservative because it must rely on our contemporary imaging methods for visualizing the brain. Imaging methods are almost certain to improve in the future, thereby improving inference of the original state of damaged neural structures. If brain preservation meeting verifiable criteria were the only option allowed, then lethally injured people who might otherwise have a chance at future recovery would be unable to access potentially life-saving procedures.

Instead of verifiable brain preservation, we can imagine a brain preservation procedure that has the *potential* to preserve valued aspects of psychological information. Such procedures are available today. In this review, we propose that a reasonable option given our currently available technology is to make our best effort to determine what are the necessary structural components of valued information in the brain and attempt to preserve them. We refer to this approach as “experimental brain preservation” because it involves techniques that are based on current neuroscientific theories but have not yet been proven to successfully preserve the information required for psychological properties in humans. The main distinction between verifiable and experimental brain preservation is the level of certainty in preserving the information required for psychological properties (Table 1). Verifiable preservation is defined as demonstrable retention of these properties, while experimental preservation makes a best effort to preserve them, acknowledging the uncertainties involved in our current understanding and technological capabilities. An experimental brain preservation procedure acts as a potential bridge to future medical capabilities, subject to uncertainty about its likelihood of success, rather than being a form of definite survival. Critically, while people preserved in such a manner are legally dead, they may not yet be dead according to the loss of personal identity or the information-theoretic criteria of death, which is the point at which the brain has been damaged so severely that all information it once contained about valued psychological properties such as memories can no longer be inferred (20, 21, 28–30). The question may not be binary because degrees of survival are possible (31). Given the dissatisfaction in the medical community with the current legal and clinical definitions of death (148), it is prudent to not ignore interventions which might be compatible with saving lives under plausible alternative definitions (32).

**Table 1:** Differences between verifiable and experimental brain preservation procedures.

Aspect	Verifiable brain preservation	Experimental brain preservation
Definition	A procedure that demonstrably preserves a person's psychological properties	A procedure that attempts to preserve valued aspects of psychological information, but without guaranteed success
Certainty of preservation	High—preservation of psychological properties can be verified	Uncertain but possible—unable to verify whether the procedure retains critical information, but attempts to do so consistent with our best current understanding
Basis of preservation	Known and verified structural correlates of psychological properties	Best current estimates of necessary structural components
Current availability	Not yet possible with current knowledge and technology	Available with current technology
Imaging requirements for verification	Relies on contemporary imaging methods for verification	May benefit from future improvements in imaging technology
Threshold for implementation	Strict—must meet verifiable criteria	More flexible—allows for attempts even with uncertainty
Potential for future recovery	High certainty of potential recovery	Uncertain, but provides a possible bridge to future medical capabilities
Accessibility	Limited due to strict inclusion criteria	Accessible to a wider group of individuals

## What structures in the brain need to be preserved?

If the brain required continuous neural activity for maintenance of valued psychological properties, then brain preservation would be a much more difficult problem. However, for long-term memories and personality, three pieces of evidence point against this hypothesis. First, research on *C. elegans* and rabbit hippocampal slices indicates that biological time can be paused via cryopreservation without losing correlates of long-term memory, suggesting that key aspects of cognitive function can be preserved despite temporary cessation of molecular motion (33, 34). Second, in the surgical procedure of deep hypothermic circulatory arrest, brain electrical activity ceases temporarily without major impact on long-term memory or personality (35). Finally, cases of cardiac arrest induced by hypothermia, such as in avalanche survivors, further show that extended periods without brain blood flow, while temporarily halting electrical activity, do not necessarily lead to loss of long-term memories or personality traits (36). Instead, it is the information contained within the structures that are important, while the functions of the brain can be paused and restarted. Short-term memory recall, on the other hand, is more likely to be dependent on labile functional states of brain cells, and it is less likely for there to be a current way to preserve this (37). It is critical to emphasize that contemporary brain preservation is unlikely to be able to preserve all psychological states in the brain. Instead, it is only likely to be possible to preserve information that is encoded via more stable structures, such as that required for long-term memory recall and personality traits. By structures in the brain, we refer to both individual biomolecules and their spatial relationships, which compose the morphologic features that can be measured via microscopy.

In humans, long-term memories can be accessed in less than a second in a process that involves communication between multiple brain regions that are millimeters to centimeters apart (38). A wealth of evidence suggests that the only neural process that could instantiate such a rapid and widespread process of long-term memory recall is rapid electrochemical ion flow through the connectome—i.e., the complete map of brain cell connections (39–41). While the connectome provides a morphological basis, it is very likely that certain biomolecules such as ion channels, ion pumps, and neurotransmitter receptors also play a crucial role in mediating memory recall and other cognitive functions. Thus, it is the extent of preservation of the *biomolecule-annotated connectome* that makes the most sense as a metric for evaluating the quality of a brain preservation procedure. However, contemporary preservation procedures do not require flawless maintenance of the biomolecule-annotated connectome to be potentially sufficient to retain the information required for long-term memory recall. For example, biomolecular information in the brain is largely redundant, organized into highly correlated sets of modules and sub-modules (42). Theoretically, even if some biomolecules in a module were damaged or destroyed, their approximate relative levels could be predicted to some degree of accuracy via profiling the remaining biomolecules in the module (43). Morphological information such as cell membrane shape can also be predicted through inference of the breakdown and diffusion patterns of the biomolecules that compose them, such as cell surface proteins, which can provide a unique barcode to each cell (44). Additionally, many neural structures are not completely stable over time but rather evolve during life, even as memories remain roughly intact, allowing a degree of leniency in the required precision in inference of the original states (45, 46). Therefore, the most important metric—albeit an elusive one—is our ability to infer the original states of the biomolecule-annotated connectome that are critical for the information in valued psychological properties.

In the 2010s, Kenneth Hayworth at the Brain Preservation Foundation (BPF) put forth a prize to develop a brain preservation technique capable of maintaining the brain's ultrastructure for at least a century (47). There were two primary criteria, assessed by electron microscopy: (a) connectome traceability, i.e., the ability to unambiguously trace neurites across sequential image sections, and (b) whether cell membranes and components such as organelles and vesicles looked as expected, judged against the established knowledge of neuroscience. Setting a high bar such as this is a very useful aspirational goal, but it risks inadvertently overlooking methods that produce damage that may still be recoverable with the aid of future technologies. Take, for instance, vacuolization, which is a common postmortem artifact (48). Vacuolization can compress neural structures, preventing their visualization via contemporary electron microscopy approaches. However, crucially, this might not significantly damage the actual information content within the biomolecule-annotated connectome (49). Similarly, synaptic or dense-core vesicles might degrade or disperse postmortem, yet the biomolecules that constitute these vesicles and dictate their organization will still be present in the local area for a window of time even after they can no longer be seen under the microscope. Future biomolecular mapping techniques could potentially still infer this information to a sufficient degree of accuracy based on visualizing the breakdown products and building a physical model of how their degradation occurred.

An alternative metric is to visualize brain tissue in multiple ways and evaluate whether each form of preservation damage present—i.e., each deviation from the expected morphology *in vivo*—is likely to indicate a true loss of information content in the biomolecule-annotated connectome. For example, fixation methods can alter the volume of the extracellular space from the *in vivo* estimate, but this can be recovered with reconstructive algorithms (50). To be clear, though, this is not an argument for complacency. Each of these structural inference methods can and should be tested in the near-term. The eventual goal should be to improve preservation methods to achieve the gold standards of connectome traceability and *in vivo* morphologic preservation quality without the need for inference. Additionally, as our knowledge of the neuroscience of memory improves in the future, our procedures for testing preservation quality should also be modified as necessary. Ideally, collaborative research should be performed with experts in memory retrieval, brain preservation, microscopy, biomolecular profiling, and other related fields, so that preservation methods are corroborated and improved in an iterative process over time.

## Contemporary methods for brain preservation

**Figure 1:**
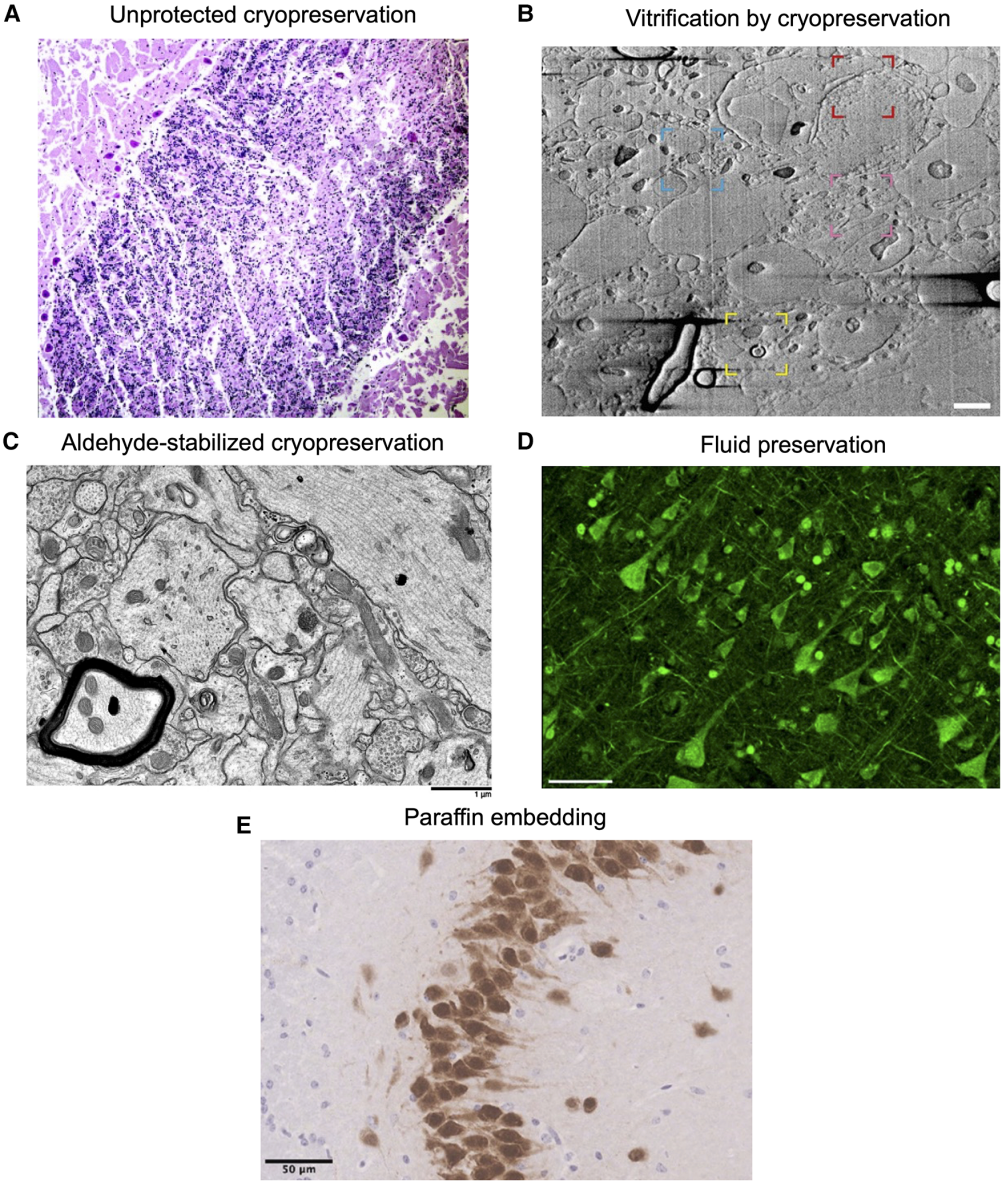
Example images showing morphology preservation in brain tissue preserved with different methods. **(A)** Image from (56). Crisscross linear clefts due to ice artifact in bovine cerebellar tissue, because of freezing and thawing without cryoprotectant. **(B)** Image from (57). Focused ion beam scanning electron microscopy (FIB-SEM) of a 200 µm brain tissue section cryoprotected in 20% bovine serum albumin and vitrified using high-pressure freezing, demonstrating preservation of myelin (yellow region), potential nuclear pore complexes (red), mitochondria (blue), and a synapse (pink). Scale bar: 1 µm. **(C)** Image from (53). Electron microscopy of rabbit brain fixed with 3% glutaraldehyde, cryoprotected with 65% ethylene glycol, vitrified, rewarmed, and cryoprotectant removed, demonstrating well-preserved structures. Scale bar: 1 µm. **(D)** Image from (58). Formalin-fixed human cortical tissue stained for pan-axonal neurofilaments with SMI312 after storage in fixative for 25 years, demonstrating intact neurons. Scale bar: 50 µm. **(E)** Image from (59). Perfusion-fixed mouse brain tissue that was dissected, paraffin embedded, and stained with NeuN, demonstrating expected neuronal morphology. Scale bar: 50 µm. All images reproduced under a Creative Commons license, available here: https://creativecommons.org/licenses/by/4.0/.

Among other factors, our estimates for the time that will be necessary to wait while in preservation depends on how long it is expected to take for restoration technology to be developed, if this ever becomes possible. Opinions on this will vary significantly. Following the BPF prize criteria, 100 years of storage could be considered a reasonable initial goal. We delineate five categories of methods that could potentially preserve the brain for this amount of time, each with upsides and downsides (Table 2; Figure 1).

**Table 2:** Procedural description, examples, and trade-offs among classes of structural brain preservation methods.

Method	Procedure	Upsides	Downsides
Unprotected cryopreservation	• Sub-zero cooling of brain tissue in the absence of cryoprotectants • Variations: cooling rate, storage temperature • Ex: (51)	• Widely available initial preservation procedure • Biomolecule distributions will be altered, but minimal direct chemical changes	• Inevitable ice damage causes morphologic artifacts • Any unplanned rewarming would damage cell morphology • Long-term storage requires significant cost
Cryopreservation with CPAs	• Cryoprotectants perfused during cooling, potentially allowing vitrification • Variations: CPAs used, cooling rate • Ex: (52)	• After CPAs are removed, tissue microstructure looks intact • CPAs tend to minimally alter biomolecules • Plausibly on the shortest path to suspended animation	• Cryoprotectant toxicity • Relies on high-quality perfusion to avoid ice damage, which can be challenging postmortem • Long-term storage requires significant cost
Fixation and cryopreservation	• Chemical fixation and subsequent CPA-based cryopreservation • Variations: CPAs perfused or immersed • Ex: (53)	• Connectome preservation by vitrification after fixation shown in mammals (Brain Preservation Foundation, 2018) • Fallback of chemical preservation if low temperature storage fails	• Can be reliant on perfusion quality • Long-term storage requires significant cost • Reversing crosslinks is well beyond our current technology (also applies to all methods below)
Fluid preservation	• Fixation, then long-term storage in a liquid preservative solution • Variations: fixation method, chemicals used, storage temperature • Ex: (54)	• Simple and inexpensive • Widely used with a large infrastructure in place • Appears to retain morphology and classes of biomolecules for decades	• Chemical reactions over time will alter biomolecules • Some degree of loss of biomolecules that are not directly crosslinked • Possible storage artifacts
Polymer embedding	• Fixation, then processing and embedding • Variations: paraffin, epoxy, polyester, etc. for embedding • Ex: (55)	• Can have excellent ultrastructural preservation • Minimal chemical reactions occur during storage	• Biomolecular extraction during embedding procedures • Challenging to perform on human brain scale without sectioning first • Can be expensive

CPA, cryoprotective agent; Ex, example.

Cryopreservation without cryoprotectants (i.e., “unprotected” cryopreservation) is a widely available and easy to perform method, but it leads to unavoidable ice damage and associated morphologic artifacts, and for this reason it is not favored for morphologic preservation in brain banking (51). Ice formation not only causes morphological damage but also significant alterations to biomolecules. These alterations include changes in biomolecule location, as the mechanical effects of ice can tear cell membranes, leading to leakage of intracellular contents (60). Furthermore, ice formation can induce conformational changes in proteins by disrupting their hydration shells (61). In cryonics, researchers generally perfuse cryoprotectants in order to mitigate ice damage, which in some cases can be entirely prevented, causing the brain to convert to a glassy or vitrified state (33, 52, 62, 63). Cryonics can be considered a type of brain preservation that uses cryopreservation. The use of cryoprotectants in the cryopreservation of the brain has been explored in several studies, alongside measurements of histologic outcomes, with different outcomes (Table 3).

**Table 3:** Selected cryoprotective agents that have been used for structural brain preservation and their reported effects.

Cryoprotective agent	Biospecimen preserved and CPA delivery method	Reported histologic outcome
15% glycerol	Perfusion-based cryoprotection of cat brains	Close to normal cell arrangements with Nissl staining (64)
10% DMSO	Immersion cryoprotection of rat embryonic brain tissue	Histologically normal-appearing tissue with cresyl violet staining (65)
M22 vitrification solution	Perfusion-based cryoprotection of rabbit brains	Shrunken but reportedly preserved cells, images difficult to interpret (52)
VM3 vitrification solution	Immersion cryoprotection of thin rat hippocampal slices	High-quality ultrastructure essentially equivalent to controls, with adequate uptake of CPA in the vitrification procedure (66)
13% glycerol, 13% DMSO	Perfusion-based cryoprotection of rat brains	Preserved synaptic immunostaining, fainter NeuN staining, shrinkage of neurons (63)

DMSO, dimethyl sulfoxide; M22 and VM3, vitrification solutions composed of multiple cryoprotectants; NeuN, neuronal nuclei, a neuronal marker protein; CPA, cryoprotective agent.

Notably, the effectiveness of cryopreservation depends not only on the formulation of cryoprotectant but also on the overall procedure in which it is distributed to the brain tissue. High concentrations of cryoprotectants must be introduced gradually in a graded fashion to minimize osmotic damage. As a result, cryopreservation protocols are complex, involving optimization of cooling and warming rates, as well as the precise management of cryoprotectant concentration gradients (67). While cryoprotectant perfusion has shown promise for structural preservation in thin brain tissue samples (57), the 3D ultrastructure of whole brains after cryopreservation with perfusion of cryoprotectants, such as would be assessed with volumetric electron microscopy, has not yet been characterized in the scientific literature. This is an important area for future research.

The use of fixation followed by cryopreservation combines two powerful preservation methods, which may be helpful for maintaining structural stability over the long-term in case one of them is unsuccessful. In the published procedure of aldehyde-stabilized cryopreservation (ASC), perfusion of the chemical preservative glutaraldehyde and the blood-brain barrier modifier sodium dodecyl sulphate is followed by perfusion of the cryoprotectant ethylene glycol (53). The use of ASC to preserve an intact pig brain was judged to have met the Brain Preservation Prize's requirement of electron microscopy-based connectome preservation quality, which was awarded in 2018 (68). However, this same level of whole connectome preservation quality has not yet been demonstrated in a human brain using this method. The mechanism through which aldehyde fixation mitigates structural damage during cryopreservation with cryoprotectants is not fully established, but likely involves (a) stabilizing membranes to mitigate damage due to dehydration and osmosis (69), (b) stabilizing blood vessels to improve cryoprotectant perfusion, and/or (c) increasing the cellular permeability of cryoprotectants. Biochemically, fixation with glutaraldehyde rapidly cross-links biomolecules within minutes, retaining most cytoplasmic proteins in place (70). This is obviously highly toxic to cells and is expected to kill cells by contemporary metrics of viability. On the other hand, glutaraldehyde fixation is not expected to cause the direct loss of most macromolecules from brain tissue (71). Studies of sample preparation for electron microscopy have instead found that biomolecular extraction primarily occurs during subsequent processing steps, especially dehydration (72). However, the conformation of biomolecules can be altered due to crosslinking by glutaraldehyde (73). Finally, it is worth noting that small molecule distributions, such as electrochemical gradients across cell membranes, are likely to be altered following any method of extracorporeal perfusion, as also occurs in the reversible surgical procedure of total body washout (74). This alteration of small molecule distributions is a shared limitation across perfusion-based brain preservation procedures, including ASC.

In the polymer embedding method, fixation is performed and then the brain is processed for embedding in a material that can solidify, such as paraffin or a type of resin (55). There are numerous embedding agents and procedures that could potentially be used for preserving the structure of the brain (Table 4) (75–79). These include traditional paraffin embedding, more specialized techniques with epoxy or acrylic resins that are typically used for electron microscopy, as well as embedding agents are commonly used for plastination, such as silicone, epoxy, or polyester (80). Although polymer embedding can lead to high-quality morphologic preservation, it generally requires the extraction of lipids and is challenging to perform on specimens the size of the human brain without distortions. Because of limitations in their incubation times, some resins might require the brain tissue to be sectioned into smaller pieces before embedding, which would lead to damage at the cut interfaces (81). Other techniques, like plastination, have shown promise for embedding larger specimens, including whole organs. But even when using plastination methods, brain tissue is usually cut into sheets prior to embedding (82). The degree of ultrastructural preservation achieved in plastination is also uncertain, especially for the brain, and especially when the tissue is dehydrated at −25°C, which can cause damage due to ice crystal formation (83). A key advantage of polymer embedding methods is that they have the potential to allow for preservation over very long timescales without any required upkeep. This is particularly true for epoxy resins, which are thought to have excellent long-term stability following the crosslinking polymerization reaction (84). Because the degree of ultrastructural preservation and biomolecular retention can vary significantly between different polymer embedding methods, and they have not been widely tested on tissues the size of the whole human brain, careful consideration and further research would be indicated if one were designing a polymer embedding procedure for brain preservation.

**Table 4:** Upsides and downsides of potential embedding agents for brain preservation.

Embedding agent	Upsides	Downsides
Paraffin	• Widely used, relatively inexpensive • Long-term preservation data available • Protein antigens well-preserved • Can be performed on large sample volumes	• Necessitates removal of lipids (applicable for all, to some degree) • Unclear degree of ultrastructure preservation • To our knowledge, no volumetric electron microscopy data is available for paraffin embedded brain tissue • Causes tissue shrinkage
Celloidin	• Decreased need for heating, less tissue shrinkage • Possibly better preservation of internal structures than paraffin	• Very long infiltration times, months for large tissues • Blocks must be maintained in liquid ethanol • Flammable, potentially dangerous
Epoxies	• Standard method used for high-quality ultrastructure preservation • Easy to verify preservation quality with electron microscopy • Potential for very long-term stability with storage at ambient temperature	• Highly viscous resins, require long and high temperature infiltration • Can damage protein antigenicity • No established protocols for embedding specimens the size of human brain, so would require sectioning prior to embedding
Acrylates	• Some allow for good tissue infiltration of large samples • Can polymerize at low temperature • Can have high ultrastructural preservation • Often better biomolecular preservation	• Challenging to cure large samples via UV radiation • Often reported to not yield as high of ultrastructure preservation quality as epoxy resins • Questions about long-term stability, e.g., may require storage in desiccator
Plastination agents	• Has been used to preserve whole brains and even whole bodies • Methods designed to be relatively cheap and widely accessible • • Protocols often include or can be adapted for use with epoxy or polyester resins	• Histological preservation is poorly characterized, as this is usually not the goal • Some protocols still require sectioning brains into slices • Can cause substantial shrinkage • Very long-term stability unestablished

## Rationale and steps of whole brain fluid preservation

We next discuss fluid preservation in-depth. In the method of fluid preservation, the initial fixation is performed, then the brain is stored long-term in a liquid preservative solution. This method has several advantages. There is a large infrastructure in place worldwide to perform the relevant procedures, due to overlap with the fields of pathology and brain banking. It is simple, which is important because complexity presents its own risks. It is broadly considered a good method for morphologic preservation (54, 85). It is by far the most well-studied and commonly practiced method of banking whole human brains for histology studies, with tens of thousands of brains banked in this manner across the world (86, 87). Because the cost is so low, we could easily envision a time when philanthropic funds are able to pay for this procedure for all those who desire it. Therefore, it deserves serious consideration as a brain preservation method. Notably, by discussing this method, our goal is certainly not to disparage other preservation methods, which we consider to be worthy of significant further research as well.

**Figure 2:**
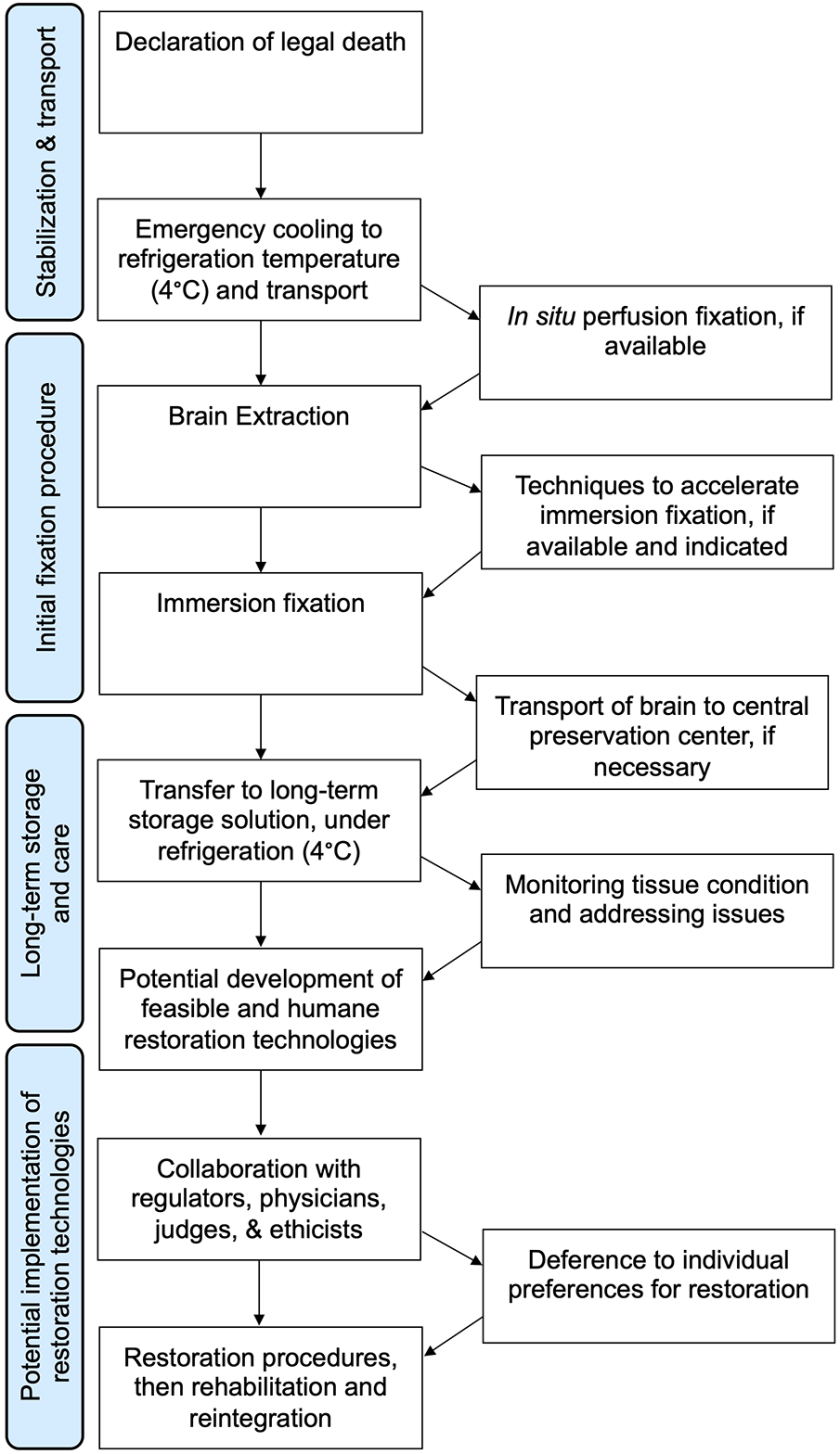
Flowchart for one way of implementing a whole brain fluid preservation procedure with the goal of potential restoration.

We present a basic flowchart for the steps involved in whole brain fluid preservation with the goal of potential recovery (Figure 2). The first step is stabilization and transport. Any postmortem delay prior to fixative reaching brain tissue should be minimized as much as possible. However, available evidence suggests that the biomolecule-annotated connectome does not degrade immediately, but rather decomposes over a timescale of hours to days (48, 88). This is especially the case if the brain is cooled to refrigeration temperatures of about 4°C, which should be initiated as soon as possible. This should not imply that procedures should not be carried out with the utmost urgency, but that we should be hesitant to forgo preservation efforts unless there is clear evidence of complete neural structure degradation.

Perfusing fixatives by using pressure to drive liquid preservatives through the cerebrovascular system has the potential to distribute chemicals rapidly across the brain and is considered the “gold standard” for preservation in laboratory animals (89–91). Despite the no-reflow phenomenon, many investigators have reported that perfusion after hours of postmortem delay is still useful to help distribute fixatives (90). How rapidly cerebral perfusion degrades in the postmortem period, and how this can be mitigated, is an open question worthy of future research efforts. Regardless of whether perfusion is available and successful, a subsequent step in the algorithm is brain extraction. Although extraction can potentially be a source of damage, this damage can be minimized through the use of careful technique (92, 93). Indeed, most of our accumulated knowledge of human neurohistology has come from brains that have been extracted from the skull. Traumatic handling of the brain can introduce known artifacts such as “dark neurons”, but this artifact likely result from a reversible gel-gel phase transition, and is not expected to cause discontinuities in the plasma membrane that would prevent connectome inference (94, 95).

The next step in the procedure is immersing the brain in a preservative fluid that penetrates from the surface inward. An immersion step is used because perfusion alone is not reliable in many cases, even in ideal laboratory animal experiments (96). Alternatively, if perfusion is not available, then immersion fixation is the only option. The main problem with immersion fixation is that the depth of fluid penetration is proportional to the square root of time, so inner brain regions will undergo a degree of decomposition before fixative reaches the tissue (97). It may take weeks or months before immersion fixation is complete, during which cellular structure may be degrading (98). Consistent with this, some investigators report decomposition in the inner brain regions of immersion fixed brains (90, 99). On the other hand, other studies report acceptable histologic quality in inner brain regions following an adequate amount of time for immersion fixation (100, 101). This is especially the case in studies that make use of the brain's ventricular system to help distribute fixative during immersion (102, 103). The differences between studies may be partly due to the use of refrigeration, which slows down decomposition during immersion fixation to a substantial degree (104). Also, this may be partially a question of what metric different studies are using to evaluate the resulting histology. Research using more quantitative metrics of histology quality and evaluation of larger samples of brain tissue are needed to better address outstanding questions about the quality of immersion fixation. For example, total equivalent normothermic ischemia for different areas of the brain during immersion fixation can be calculated by a measure called the S-MIX (Standardized Measure of Ischemic Exposure), which can be correlated with expected structural changes at different timepoints of ischemic exposure (105).

Following fixation comes a potential transfer to a storage solution that is optimized for long-term preservation, preferably at low, but non-freezing temperatures. The only reason that fluid preservation is a plausible long-term brain preservation method is that crosslinking fixation alone is an extremely powerful preservation method (106, 107). The topic of fluid preservation was recently the subject of a comprehensive review (108). Briefly, fluid preservation dramatically strengthens native gel-like networks in cells and the extracellular matrix, effectively converting the brain into a series of interconnected chemical gels (109). The initial fixation procedure largely crosslinks proteins, but over time the fixation process retains a larger set of biomolecules, which is likely why profiling studies suggest that biomolecular content can be retained for years (110–112). Morphologic features on microscopy have also often been reported to be preserved for at least several decades (85, 113, 114). On the other hand, a minority of studies have identified morphological storage artifacts, which may be related to a long-term drift of non-crosslinked molecules such as a subset of lipids (115, 116). Fluid preservation can be enhanced through mitigating molecular drift by increasing the viscosity of the maintenance fluid, such as by adding glycerol (117). An alternative option is to perform tissue clearing prior to long-term preservation (118). This would remove the lipids in the brain, but offer several advantages, including repeated non-invasive imaging, and potentially reduced oxidative damage over time (119). It is plausible that there are multiple fluid preservatives that could accomplish the same goal of retaining enough structural information in the biomolecule-annotated connectome over the long-term, but this is not well established, and more research on the topic is needed (120). If in the future it is determined that a very long period of storage of the fixed brain in a liquid solvent leads to significant damage to the biomolecule-annotated connectome, then it would be possible to switch the brains preserved in liquid to a different preservation method, such as cryopreservation or polymer embedding.

## Effects of preservation methods on key brain structures

The biomolecule-annotated connectome can be conceptualized as having two primary components: the morphological structures that define the connectome itself, and the biomolecules that annotate these structures and mediate their functions. The most critical morphological aspect of the connectome is the shape of cell membranes. This defines neuronal and glial boundaries, including those of specialized structures such as synapses, dendrites, axons, myelin, and astrocyte processes that enable cell-to-cell communication. These structures collectively define the physical “wiring diagram” of the brain and dictate the paths that electrochemical signals take as they propagate through neural circuits. Importantly, cell membranes share relatively similar biomolecular compositions, so evaluating the morphologic preservation quality of one type of cell membrane provides insight into how other types of cell membranes are also likely to be preserved with a given procedure. Annotating these morphological structures are several key classes of biomolecules. The key informational features of biomolecules are their relative location, their atomic composition, and their conformations. Proteins, such as receptors, ion channels, scaffolding proteins, and enzymes, are critical for mediating electrical and chemical signal transmission. Lipids, the primary constituents of cell membranes and myelin, also affect ion flow through several mechanisms, including by modulating protein function (121). Nucleic acids, including genomic DNA and various RNA species, while not directly affecting rapid ion flow, can play an important role as a source of information about cell function if other structures are damaged. Notably, a DNA-associated innate immunity pathway has been found to play a role in memory formation, suggestive of additional roles that nucleic acids can play in cognition (122). Together, these biomolecules influence the functional properties of the connectome and mediate the dynamic activity patterns that are thought to underlie memory recall and other forms of cognition. Although labile small molecules and ions themselves are clearly also critical for ion flow, their distributions can be lost in certain situations without loss of stored long-term memories, for example, in cortical spreading depression or temporary cerebral ischemia (123, 124). The more stable macromolecules appear to be more critical to preserve. Therefore, the “parts list” we focus on as our current best guess for the key components of the biomolecule-annotated connectome are the cell membrane morphologies and the proteins, lipids, and nucleic acids that annotate them.

For each of the brain preservation methods described, we can estimate how they would preserve each of these components of the biomolecule-annotated connectome. We focus on a hypothetical ideal case, where the procedure is started immediately at the time of legal death without any atypical impairments to perfusion. The expected preservation quality in non-ideal cases may differ and depends on the specific deviations from the ideal scenario. With the notable exception of unprotected cryopreservation, each preservation method has different relative strengths and limitations (Table 5).

**Table 5:** Summary of expected preservation effects on key components of the biomolecule-annotated connectome.

Structural feature	Unprotected cryo-preservation	Cryo-preservation with CPAs	Fixation and cryo-preservation	Fluid preservation	Polymer embedding
Cell membrane shape	• Expected to be lost in many areas throughout the brain due to ice damage	• Could be retained, but ultrastructural preservation is unproven in whole mammalian brains	• Found to preserve the connectome via electron microscopy in the pig brain • Not yet shown in human brains	• Largely expected to be intact, with certain artifacts found over time in some studies (108)	• Largely expected to be intact using epoxy methods • But currently unable to infiltrate whole brain with epoxy
Proteins	• Mostly present • May aggregate, move, or change conformation with ice damage	• Mostly present • May have conformation changes due to CPAs, but likely minimal	• Mostly present • Both location and composition expected to be unchanged • Conformation may be altered	• Mostly present • May be change to chemical composition • Conformation may be altered	• Mostly present • Harsh solvent treatment could affect conformation and composition
Nucleic acids	• Mostly present, but may be damaged due to ice formation	• Mostly present, not expected to have major changes	• Mostly present • Molecules are fixed, so unable to dissociate them for DNA sequencing	• Mostly present • May accumulate damage over time, unclear degree	• Likely mostly present • Uncertain effects of processing on DNA content
Lipids	• Mostly present, but may be damaged due to ice formation	• Mostly present, not expected to have major changes	• Mostly present, not expected to have major changes	• Mostly retained but inaccessible • Subset may be lost or chemically altered	• Subset retained due to osmium fixation • But many lipids are expected to be extracted

Although unprotected cryopreservation has the ostensible upside of not inducing any damage due to exogenous chemicals, this is misleading because the inevitable ice formation when cryopreserving whole human brains in this manner would lead to substantial damage to both individual biomolecules and the morphological features they compose. Cryopreservation with CPAs, on the other hand, shows promise for structural preservation. Additionally, it does not induce any biomolecular alterations due to crosslinking. However, the ultrastructural-level preservation quality following cryopreservation with CPAs is unproven in whole mammalian brains. All methods using chemical fixation offer excellent initial morphologic preservation. Fixation combined with cryopreservation in the procedure of ASC is the only technique that has been shown to preserve the connectome of a mammalian brain, alongside an accepted argument that this storage approach could maintain the preservation for at least 100 years (68). Fluid preservation has been found to maintain most structures studied, but can introduce chemical alterations to biomolecules over time, requiring further research to determine the effects of these changes on key neural structures over long periods. Polymer embedding, particularly with epoxy resins, shows excellent potential for ultrastructure preservation. However, it causes lipid extraction and cannot be applied to whole brain specimens, thereby requiring sectioning and leading to cutting damage. It is important to note that this is a highly dynamic field. As a result, this summary of preservation quality should be considered preliminary and is expected to be updated in the future as our knowledge of brain preservation improves.

The preservation outcomes in this table are what might be expected in a hypothetical ideal procedure started immediately at the time of legal death. Note that this table represents a generalized overview, and the actual preservation quality may vary depending on the specific implementation of each method. The information presented is preliminary and likely to be updated as more experiments are performed and our knowledge of brain preservation improves.

## Potential future restoration technologies

Regardless of the structural brain preservation method used, there are two major classes of restoration methods that have been proposed over the years: molecular nanotechnology-based approaches and whole brain emulation approaches. Regarding nanotechnology approaches, the first step would likely involve detailed molecular imaging and modeling, which would allow computer-based inference of the most likely original states of the biomolecules and guide the restoration procedure (125). Notably, the major extant nanotechnology approaches that have been proposed for repair following brain preservation via cryopreservation have stipulated that it would also be possible to repair aldehyde crosslinks, similar to other forms of molecular damage occurring in brain preservation (19, 20, 126). Such a technology would need to not only sense the chemical bonds formed by an aldehyde crosslink, but also to sense the broader chemical milieu so as to recognize that it is an artificial link between biomolecules, and thereby distinguish it from any such bonds that also occur *in vivo*. At that point, the crosslinking bonds could be cut, and the aldehyde removed. Of course, this is impossible today and any such future technology is quite far away. Moreover, we do not yet even have a full understanding of the molecular mechanisms of aldehyde crosslinking (127). However, crosslinks are already ubiquitous in our cells and able to be repaired via reactions catalyzed by endogenous enzymes, emphasizing that their removal is clearly physically possible (128, 129). Conditional on such advanced nanotechnology being available, which is highly uncertain, the key question for recovery will likely be the degree to which valued structural information such as that mediating memories and personality is preserved.

The other most frequently discussed restoration strategy is whole brain emulation. In one version of this method, the brain tissue would first be processed, sectioned, and imaged in detail at the molecular level (130). Next, software would reconstruct the original state of the brain prior to damage due to the dying and preservation processes. Finally, the person would either be revived with a machine body to operate in our physical world or with a digital body in a digital world. A major concern with whole brain emulation is that people are concerned about losing control over one's body autonomy and becoming indefinitely trapped in an undesirable or even abusive situation. This is an understandable concern deserving of serious consideration. However, in our view, this would require societal collapse or a dramatic regression of protections for civil rights, which would also affect any humans living at the time, making it a generalized argument against any form of potential life extension. Absent dystopian changes to society, any realistic restoration procedure in a civilized society will be highly regulated to ensure that the revived individual retains control over their body autonomy.

It is critical to note that the proposals for both whole brain emulation and nanotechnology are highly speculative and face numerous limitations that are far beyond our current scientific understanding and engineering capabilities. The gap between our present situation and the level of technology required for such interventions is immense and may prove insurmountable. Upon deciding to preserve their brain today, a person can choose to record their preferences for how and when the restoration process would be performed, if it ever becomes possible. In the future, any organization that performs restoration should clearly be highly regulated. The decision-making team would ideally be required to consider the individual's preferences regarding restoration to the maximal extent possible, given the technology and resources available to them.

## Legal and ethical aspects of brain preservation

Clearly, brain preservation must be performed in a way consistent with societal laws and ethical standards. We refer the interested reader to some of the many previous thorough discussions of these topics (131–135). Briefly, we will highlight several areas in which legal and ethical aspects of the field interact with the brain preservation procedure.

First, there are several forms of legal delays occurring before the procedure that can prevent people from achieving adequate preservation quality. It is clear that significant damage begins within minutes (48). Barriers to prevent the procedure from beginning include forensic investigation protocols, hospital policies, and in some cases, mandated forensic autopsy. The organ donation for transplantation field has dealt with similar problems, and in many jurisdictions has achieved a good working relationship between their interests and those of medical examiners and coroners, allowing donation to proceed in most cases (136). One source notes that there has not been any recorded cases found in medical and legal publications where the process of obtaining organs has hindered a criminal investigation (137). With enough societal interest, the brain preservation field could achieve a similar collaboration with medical examiners and coroners, to allow high-quality brain preservation without preventing adequate forensic investigation when necessary.

From an ethical perspective, it is critical for people choosing the procedure to understand that significant uncertainty surrounds the capability of current brain preservation procedures to maintain psychological information for future recovery. Given its dependence on future molecular imaging technologies, it is not currently possible to decide with certainty where the line between meaningful and futile brain preservation lies. It should also certainly not be mistaken for a form of long-term suspended animation, i.e., a procedure readily reversible with contemporary technology. It is imperative that any marketing materials, such as a website, explicitly convey these uncertainties to prevent offering false hope and inducing harm. There needs to be informed consent to ensure that people choosing the procedure are aware that any long-term outcomes are unknown. Further, the procedures must only be initiated after other interventions have either failed or been declined (138), and must conform with local laws.

Finally, it is essential to discuss some of the ethical obligations of brain preservation organizations. The initial cryonics organizations were very poorly run, leading to thawing and decomposition of the bodies and their information-theoretic death, regardless of the quality of the initial cryopreservation (139). It is essential for brain preservation organizations to maintain stability, including adequate funding, to prevent a similar tragedy in the future. It is also essential for the people working for the organizations to care for the preserved brain as a human person, not as human remains (140). Making the choice for brain preservation is a courageous and pro-social decision that benefits others, by stimulating research and decreasing social stigma around the practice. This choice needs to be respected and honored by any organization choosing to engage in brain preservation.

## Discussion

Proponents of cryonics and chemical brain preservation have been advancing arguments supporting these practices for several decades (18, 19, 52, 62, 132, 141). In recent years, there is increasing evidence from neuroscience suggesting that memories are most likely encoded in the brain's intricate structures, including its synaptic connectivity (142–144). Recent studies using novel tissue clearing methods have also corroborated the preservation of neural circuitry in brains preserved in formaldehyde for many years, thus raising the prospects for the fluid preservation method in particular (145, 146). As a result, it makes sense to seriously consider methods of experimental brain preservation as an option upon legal death as a potential bridge to health restoration technologies that may be developed in the future. Moreover, research in this area will potentially have spillover benefits to other fields, including improvements in methods to study brain disorders and neural ischemia (104), improvements in techniques for organ banking (12), and enabling human space exploration (147). While there is clearly still uncertainty about whether technology will ever develop to render restoration possible, not allowing preservation at legal death to those who are interested could mean missing a fleeting chance to potentially save lives. Therefore, despite challenges in procedural optimization and ethical considerations that must be taken into account, we believe it is a valid approach to provide this option now to those who are informed and willing. Additionally, the more people who choose to pursue structural brain preservation procedures, the more affordable, accessible, and effective they are likely to become. Given the importance of scientific validation, a primary focus should be on coordinated research efforts to improve the preservation methods and test whether they are effectively preserving the structures in the brain thought to be required for valued psychological properties to be maintained.

## Data Availability

The original contributions presented in the study are included in the article/Supplementary Material, further inquiries can be directed to the corresponding authors.
